# Nash Bargaining Game-Theoretic Framework for Power Control in Distributed Multiple-Radar Architecture Underlying Wireless Communication System

**DOI:** 10.3390/e20040267

**Published:** 2018-04-11

**Authors:** Chenguang Shi, Fei Wang, Sana Salous, Jianjiang Zhou, Zhentao Hu

**Affiliations:** 1 Key Laboratory of Radar Imaging and Microwave Photonics, Ministry of Education, Nanjing University of Aeronautics and Astronautics, Nanjing 210016, China; 2 School of Engineering and Computing Sciences, Durham University, Durham DH1 3DE, UK; 3 College of Computer and Information Engineering, Henan University, Kaifeng 475004, China

**Keywords:** cooperative game, Nash bargaining solution, power control, interference power constraint, signal-to-interference-plus-noise ratio, distributed multiple-radar system

## Abstract

This paper presents a novel Nash bargaining solution (NBS)-based cooperative game-theoretic framework for power control in a distributed multiple-radar architecture underlying a wireless communication system. Our primary objective is to minimize the total power consumption of the distributed multiple-radar system (DMRS) with the protection of wireless communication user’s transmission, while guaranteeing each radar’s target detection requirement. A unified cooperative game-theoretic framework is proposed for the optimization problem, where interference power constraints (IPCs) are imposed to protect the communication user’s transmission, and a minimum signal-to-interference-plus-noise ratio (SINR) requirement is employed to provide reliable target detection for each radar. The existence, uniqueness and fairness of the NBS to this cooperative game are proven. An iterative Nash bargaining power control algorithm with low computational complexity and fast convergence is developed and is shown to converge to a Pareto-optimal equilibrium for the cooperative game model. Numerical simulations and analyses are further presented to highlight the advantages and testify to the efficiency of our proposed cooperative game algorithm. It is demonstrated that the distributed algorithm is effective for power control and could protect the communication system with limited implementation overhead.

## 1. Introduction

### 1.1. Background and Motivation

In recent years, the explosion of wireless devices has led to a sharp increase in demand for more spectrum [1]. Thus, the radio frequency (RF) spectrum congestion has been a challenging problem that the whole world has to face. Traditionally, different wireless devices are widely separated in the frequency band such that they do not interfere with each other. However, owing to the services with higher bandwidth requirements, the traditional solutions to the RF spectrum scarcity do not work. Subsequently, various approaches such as waveform design, dynamic RF spectrum sensing, power control and radiation time management, have been exploited for spectrum sharing. It should be noted that spectrum sharing has gained remarkable interest both from academia and industry due to its potential to overcome the problem of spectrum scarcity, which is caused by the current way of fixed spectrum allocation [2]. Spectrum sharing allows two or more users (radars or communication systems) to share the same RF spectrum as long as they do not generate too much harmful interference for each other [3].

Due to its agility and adaptation capability, spectrum sharing is broadly exploited and opens up a new perspective for spectral coexistence between radar and wireless communication systems. It can be done either through the time dimension or through the space dimension [3]. In the first case, radar and communication users utilize the spectrum resource in different time slots. Pual and Bliss define the constant information radar (CIR) and propose an information-driven radar algorithm to limit radar spectrum utilization for dynamic shared spectrum access [4]. The target tracking loop period is maximized to decrease the shared spectrum impact on communication systems, while guaranteeing a specified mutual information (MI). Zhu et al. propose a collaborative spectrum sharing protocol with optimal time allocation [5], where the quality of service (QoS) of both primary and secondary users is improved. It is also shown that the sum transmission rates of primary and secondary users are maximized. The spectral coexistence of power-controlled cellular networks with rotating radar is investigated by Yin et al. [6], in which the cellular system reduces its transmit power for the period when the radar’s directional antenna main beam is pointing to it. In the second case, radio frequency (RF) devices employ the spectrum in different locations. Turlapaty et al. present a dynamic spectrum allocation algorithm for the spectral coexistence between a radar system and a communication system [7], the operating frequency bands of which overlap. Romero and Shepherd optimize the radar transmitted waveform to share the spectrum with the existing communication systems such that the radar detection performance is not compromised with the protection of the communication systems’ symbol error rates (SERs) [8]. Li et al. propose a joint design of the MIMO communication transmit covariance matrix and the MIMO radar sampling based on sparse sensing and matrix completion [9], which can minimize the interference power at the radar receiver while guaranteeing a certain average capacity. Recognizing that the precise characteristics of target spectra are impossible to capture in practice, the power minimization-based robust orthogonal frequency division multiplexing (OFDM) radar waveform design algorithm in signal-dependent clutter and coloured noise is presented [10], and it is shown that exploiting the scattering off the target due to communication signals can minimize the power consumption of the radar system. Other existing works are [11,12,13,14,15,16,17,18].

### 1.2. Literature Review

In distributed multiple-radar systems (DMRSs), game theory has been considered as a powerful tool for resource allocation [19]. Game theory models and analyses the interaction of decision makers in decentralized networks, which can be classified into two categories: non-cooperative and cooperative game models. As for the non-cooperative game, each player behaves in a selfish and rational manner to maximize its own utility as a best response to the actions of the other players. Extensive non-cooperative game-theoretic algorithms have been presented for transmit resource allocation in DMRS [20,21,22,23,24,25]. From a game theoretic perspective, Gogineni et al. develop a polarimetric waveform design approach for distributed multiple-input multiple-output (MIMO) radar target detection in the form of a two-player zero-sum game [20]. The proposed algorithm does not require training data, and it is shown that considerable performance improvement can be obtained due to the polarimetric design. The game-theoretic interaction between a smart target and a smart MIMO radar is studied by Song et al. [21], where the MI is utilized to define the utility functions and the unilateral, hierarchical and symmetric games are investigated. A non-cooperative code design scheme is proposed to maximize the SINR of each radar [22]. Bacci et al. model the radar sensors as rational players in a non-cooperative game [23], which designs the transmission power and achieves a good trade-off between target detection performance and power consumption for a radar sensor network. Deligiannis et al. address the problem of competitive power allocation for a MIMO radar in the presence of multiple targets and jammers [24], which is formulated as a non-cooperative game model to minimize the total emitted power by the radars subject to a given detection threshold for each target. Deligiannis et al. investigate a non-cooperative game theory-based power allocation strategy and perform a Nash equilibrium (NE) for a multistatic MIMO radar network [25].

However, the non-cooperative game-theoretic methods lower the system’s performance when compared to cooperative game-theoretic methods [19], which is due to the fact that each player in a non-cooperative game behaves in a selfish and rational manner to maximize its own utility. Thus, current research believes that cooperative game-theoretic models are much more suitable for resource allocation in DMRS, in which the Nash bargaining solution (NBS) is one promising candidate. Most of the existing studies concentrate on achieving the NBS for transmission power allocation. One notable study is presented [26], where a cooperative game scheme is proposed for power allocation in distributed MIMO radar networks. It is demonstrated through Monte Carlo simulations that the proposed algorithm is able to provide more accurate target localization accuracy than the uniform power allocation approach. Chen et al. developed a distributed power management method for cooperative localization in both synchronous and asynchronous networks [27], and the NBS for the power allocation in cooperative localization is derived. Further, Chen et al. extend the formulations in [26] to the target tracking scenario by adding the target kinematic model [28]. Nowadays, with the rapid development of hostile advanced interceptors, a low probability of intercept (LPI) design has been an important and essential part of military operations in modern radar systems [29,30,31]. Thus, it is necessary to minimize the radar transmitting resources while maintaining a desired radar performance. An LPI-based cooperative game-theoretic power allocation for radar networks is proposed by Shi et al. [32], which improves the LPI performance by minimizing the total transmit power for a predetermined target detection constraint. A novel signal-to-interference-plus-noise ratio (SINR)-based network utility function is defined and used as a metric to evaluate power allocation. The authors prove the existence and uniqueness of the NBS to the proposed cooperative power allocation model. It also illustrates the effects of the relative geometry configuration between target and radar networks and target radar cross-section (RCS) upon the power allocation results. Chincoli and Liotta employ machine learning to lower transmission power in wireless sensor networks [33], and it is shown that each node radiates at the minimum power level while satisfying high packet reception ratio requirements.

In view of the aforementioned works, the problem of radar and communication systems in spectral coexistence has been extensively investigated. However, it is still at an early stage, and there exist many aspects that need to be further improved: (a) all the existing works solely focus on the monostatic radar, which is not appropriate for the practical extension to the DMRS case; in the latter case, the limitations and calculations are much more complicated; (b) the game-theoretic models have not been utilized to conduct spectrum sharing between DMRS and the communication system. Labib et al. propose the idea of radar and Long-Term Evolution (LTE) systems’ coexistence by using the non-cooperative game theory [3], while as previously stated, the cooperative game-theoretic approaches can achieve a Pareto-optimal equilibrium at the Nash equilibrium (NE) point; while the effect of DMRS’s transmission on the coexisting communication system is ignored [32]. To the best of our knowledge, the problem of NBS-based cooperative game-theoretic power control for spectral coexistence of DMRS with a wireless communication system has not been investigated until now.

### 1.3. Major Contributions

Different from the existing approaches, this paper presents a novel NBS-based cooperative game-theoretic framework for power control in DMRS underlying a wireless communication system by building on the previous results in [32]. It is worth pointing out that the power allocation algorithm in [32] does not concentrate on the spectral coexistence between DMRS and the wireless communication system, and the effects of the radar transmit power on the communication user’s transmission are not analysed. Applying the idea of the NBS-based cooperative game to the spectral coexistence scenario will face many technical challenges. This is because the strategy space of DMRS not only depends on the transmit power of each radar, but also depends on the communication user’s transmission requirements. In this work, the DMRS consisted of multiple radars coexisting with a wireless communication system in the same frequency band. The aim of our work is to minimize the total power consumption of the DMRS with consideration of the protection of communication user’s transmission, target detection requirement and maximum power resource constraints. A closed-form analytical solution is obtained allowing the proposed algorithm to determine the optimal power control policy in a fast and robust manner. The comparisons with other methods confirm the advantages of our proposed scheme.

The main contributions of the present work are summarized as follows:

(1) We formulate the power control problem for DMRS underlying a wireless communication system as a cooperative Nash bargaining game, which complies fully with the Nash bargaining axioms. A unified analytical framework is proposed to maximize the overall utility function of the DMRS, where the interference power constraints (IPCs) are imposed to protect the communication user’s transmission, a minimum SINR requirement is employed to provide reliable target detection performance and the maximum power resource limitations are considered. The power control decisions of all radars are coupled in the IPCs, which makes the distributed optimization much more complex. To solve this difficulty, the IPCs are transformed into an extra pricing term in the constructed mathematical formulation [34], which not only reflects the spectrum sharing between DMRS and the communication system, but also complies with all the axioms in the Nash theorem.
(2) The existence, uniqueness and fairness of the NBS to this game are proven. Then, an iterative Nash bargaining power control algorithm is developed, which is shown to converge to a Pareto-optimal equilibrium for the cooperative bargaining game.
(3) The proposed algorithm is evaluated by extensive numerical simulations, which demonstrate that the proposed cooperative Nash bargaining power control algorithm outperforms other existing approaches in terms of power saving, target detection and spectrum coexistence performance between DMRS and communication system in the same frequency band.

### 1.4. Organization of the Paper

The rest of this paper is structured as follows. In Section 2, the system and signal models are presented. Section 3 provides the basics for the NBS-based cooperative game theory together with the cooperative power control problem formulation. Section 4 presents the solutions and algorithm implementation of the cooperative bargaining game in DMRS, while in Section 5, the performance of the proposed algorithm is illustrated via detailed comparative numerical simulations. Finally, we conclude our paper in Section 6.

## 2. System Model

**Figure 1:**
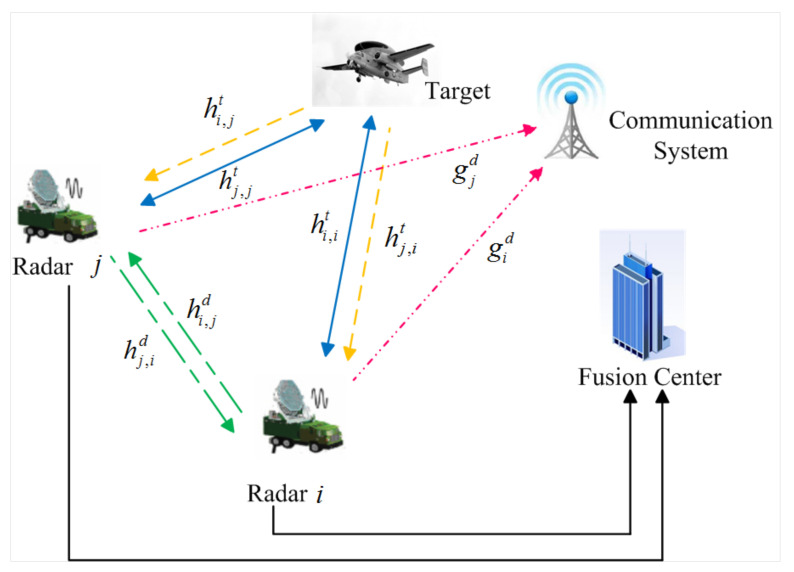
Illustration of the system model for spectrum sharing between DMRS and the wireless communication system with their corresponding channel gains.

In the present work, we consider a DMRS consisting of $M_{T}$ radars coexisting with a wireless communication system in the same frequency band, as depicted in Figure 1. A fusion centre controls the available power resources of the radars and avoids the transmission interference with the communication user. The main goal of DMRS is to minimize the total transmit power of DMRS with the protection of wireless communication user’s transmission, while maintaining each radar’s target detection requirement.

### 2.1. Problem Scenario

This subsection presents the spectrum sharing scenario. The *i*-th radar receives the echoes from the target due to its transmitted signals, as well as the signals from the other radars, both scattered off the target and through a direct path. The waveforms emitted from different radars may not be orthogonal because of various reasons, including the absence of radar transmission synchronization [35], which could induce considerable mutual interference. It is assumed that the successive interference cancellation (SIC) technique can be utilized at each radar to remove both direct and target scattered communication signals from the observed signal [16]. At the communication system, we assume that the radar transmitted signal scattered off the target is much weaker than that coming through the line of sight path from the radar transmitter, which is ignored for simplicity.

### 2.2. Signal Model

This subsection describes the signal model and presents system parameters utilized in the following. In the considered cooperative Nash bargaining game-theoretic framework, each radar performs target detection autonomously and sends its received target signals to the fusion centre, which makes a decision once the information coming from all the radars is collected. It is assumed that each radar can determine the presence of a target by employing a binary hypothesis testing on the received signal based on the generalized likelihood ratio test (GLRT) [25,32]. Thus, the *N* time-domain samples of the received signals for radar *i*, with $H_{0}$ corresponding to the target absence hypothesis and $H_{1}$ corresponding to the target presence hypothesis, can be given by:

(1) $$\left\{\begin{array}{c} H_{0}:s_{i}=\sum_{j=1,j\neq i}^{M_{T}}\zeta_{i,j}\sqrt{P_{j}}x_{j}+n_{i},\\ H_{1}:s_{i}=\chi_{i}\sqrt{P_{i}}x_{i}+\sum_{j=1,j\neq i}^{M_{T}}\zeta_{i,j}\sqrt{P_{j}}x_{j}+n_{i}, \end{array}\right.$$

where $x_{i}=\phi_{i}a_{i}$ denotes the transmitted waveform from radar *i*, $a_{i}=\left[1,e^{j2\pi f_{D,i}},\cdots ,e^{j2\pi \left(N-1\right)f_{D,i}}\right]$ denotes the Doppler steering vector of radar *i* with respect to the target, $f_{D,i}$ is the Doppler shift associated with the radar *i*, *N* is the number of received pulses in the time-on-target and $\phi_{i}$ is the predesigned waveform transmitted from radar *i*. $\chi_{i}$ represents the channel gain at the direction of the target; $P_{i}$ is the transmit power of radar *i*; $\zeta_{i,j}$ stands for the cross gain between radar *i* and *j*; and $n_{i}$ denotes a zero-mean white Gaussian noise with variance $\sigma_{n}^{2}$. It is assumed that $\chi_{i}∼\mathrm{CN}\left(0,h_{i,i}^{t}\right)$, $\zeta_{i,j}∼\mathrm{CN}\left(0,c_{i,j}\left(h_{i,j}^{t}+h_{i,j}^{d}\right)\right)$ and $n_{i}∼\mathrm{CN}\left(0,\sigma_{n}^{2}\right)$, where $h_{i,i}^{t}$ represents the variance of the channel gain for the radar *i*-target-radar *i* path, $c_{i,j}h_{i,j}^{t}$ represents the variance of the channel gain for the radar *i*-target-radar *j* path, $c_{i,j}h_{i,j}^{d}$ represents the variance of the channel gain for the direct radar *i*-radar *j* path and $c_{i,j}$ denotes the cross-correlation coefficient between the *i*-th radar and *j*-th radar.

It is assumed that channel gains remain constant during each transmission frame, and perfect knowledge of channel state information is supposed to be available to the radars in DMRS. As for the channel state information between each radar and communication user, it can be accurately estimated by the radars during the listening phase and fed back to the radar transmitters. Let us define the propagation gains of the corresponding paths as follows:

(2) $$\left\{\begin{array}{c} h_{i,i}^{t}=\frac{G_{t}G_{r}\sigma_{i,i}^{\mathrm{RCS}}\lambda^{2}}{{\left(4\pi \right)}^{3}R_{i}^{4}},\\ h_{i,j}^{t}=\frac{G_{t}G_{r}\sigma_{i,j}^{\mathrm{RCS}}\lambda^{2}}{{\left(4\pi \right)}^{3}R_{i}^{2}R_{j}^{2}},\\ h_{i,j}^{d}=\frac{G_{t}^{{}^{\prime }}G_{r}^{{}^{\prime }}\lambda^{2}}{{\left(4\pi \right)}^{2}d_{i,j}^{2}},\\ g_{i}^{d}=\frac{G_{t}^{{}^{\prime }}G_{c}\lambda^{2}}{{\left(4\pi \right)}^{2}d_{i}^{2}}, \end{array}\right.$$

where $h_{i,i}^{t}$ represents the propagation gain for the radar *i*-target-radar *i* path, $h_{i,j}^{t}$ represents the propagation gain for the radar *i*-target-radar *j* path, $h_{i,j}^{d}$ represents the direct radar *i*-radar *j* path and $g_{i}^{d}$ represents the direct radar *i*-communication system path. $G_{t}$ is the radar main-lobe transmitting antenna gain; $G_{r}$ is the radar main-lobe receiving antenna gain; $G_{t}^{{}^{\prime }}$ is the radar side-lobe transmitting antenna gain; $G_{r}^{{}^{\prime }}$ is the radar side-lobe receiving antenna gain; and $G_{c}$ is the communication receiving antenna gain. $\sigma_{i,i}^{\mathrm{RCS}}$ is the RCS of the target with respect to the *i*-th radar; $\sigma_{i,j}^{\mathrm{RCS}}$ is the RCS of the target from radar *i* to radar *j*; λ denotes the wavelength; $R_{i}$ denotes the distance from radar *i* to the target; $R_{j}$ denotes the distance from radar *j* to the target; $d_{i,j}$ denotes the distance between radar *i* and radar *j*; $d_{i}$ denotes the distance between radar *i* and the communication system.

Here, the generalized likelihood ratio test (GLRT) is used to determine the appropriate detector [25,32]. The probabilities of detection $P_{D,i}\left(\lambda_{i},\gamma_{i}\right)$ and false alarm $P_{\mathrm{FA},i}\left(\lambda_{i}\right)$ are:

(3) $$\left\{\begin{array}{c} P_{D,i}\left(\lambda_{i},\gamma_{i}\right)={\left(1+\frac{\lambda_{i}}{1-\lambda_{i}}\cdot \frac{1}{1+N\gamma_{i}}\right)}^{1-N},\\ P_{\mathrm{FA},i}\left(\lambda_{i}\right)={\left(1-\lambda_{i}\right)}^{N-1}, \end{array}\right.$$

where $\lambda_{i}$ is the detection threshold and *N* is the number of received pulses in the time-on-target. $\gamma_{i}$ denotes the SINR received at the *i*-th radar, which can be given by:

(4) $$\begin{array}{c} \gamma_{i}=\frac{h_{i,i}^{t}P_{i}}{\sum_{j=1,j\neq i}^{M_{T}}c_{i,j}\left(h_{i,j}^{d}P_{j}+h_{i,j}^{t}P_{j}\right)+\sigma_{n}^{2}}=\frac{h_{i,i}^{t}P_{i}}{I_{-i}}, \end{array}$$

where $I_{-i}$ denotes the total interference and noise received at the *i*-th radar, that is,

(5) $$I_{-i}=\sum_{j=1,j\neq i}^{M_{T}}c_{i,j}\left(h_{i,j}^{d}P_{j}+h_{i,j}^{t}P_{j}\right)+\sigma_{n}^{2}.$$

To guarantee its target detection performance, the received SINR of a radar *i* should be no smaller than a predetermined minimum value denoted by $\gamma_{i,\min}$. Thus, we obtain a target detection condition as:

(6) $$\gamma_{i}\geq \gamma_{i,\min}.$$

Finally, suppose that the transmit power of each radar is limited by $P_{i,\max}$ and that the total power consumption of all radars together is limited by $P_{\mathrm{tot}}$. Hence, the constraints:

(7) $$\left\{\begin{array}{c} 0\leq P_{i}\leq P_{i,\max},\\ \sum_{i=1}^{M_{T}}P_{i}\leq P_{\mathrm{tot}}. \end{array}\right.$$

In this study, a DMRS is allowed to coexist with a wireless communication system in the same frequency band provided that the degradation induced on the QoS of the communication system is tolerable. It is crucial to impose IPCs in the form of either global or individual constraints to control the interference generated by the radars [1].

The global IPC is utilized to prevent the total aggregate interference generated by all radars to the communication user from exceeding a predetermined threshold $T_{\max}$. Then, the global IPC can be expressed as:

(8) $$\begin{array}{c} \sum_{i=1}^{M_{T}}g_{i}^{d}P_{i}\leq T_{\max}, \end{array}$$

where $T_{\max}$ is the maximum global interference power limit prescribed by the communication system.

The individual IPC is imposed at each radar to limit interferences radiated to the communication user. This constraint is suitable for a distributed architecture, in which radars are not permitted to exchange any signalling. The individual IPC can be written as:

(9) $$\begin{array}{c} g_{i}^{d}P_{i}\leq T_{i,\max}, \end{array}$$

where $T_{i,\max}$ denotes the maximum interferences allowed by the communication system from the *i*-th radar.

## 3. Nash Bargaining Game-theoretic Power Control in DMRS

In this section, we present methodologies on the design of a game-theoretic bargaining framework for DMRS coexisting with a wireless communication system. We also review the basic definitions and concepts of cooperative bargaining games and their applications in power control problems. Moreover, the problem formulation is developed based on the bargaining games.

### 3.1. Basis of the Technique

Mathematically, the cooperative game-theoretic power control for DMRS coexisting with a wireless communication system can be described as a problem of minimizing the total power consumption of DMRS with the protection of communication user’s transmission subject to a given SINR requirement for target detection and some power resource constraints. Since the NE in a non-cooperative game is not always efficient, we resort to cooperative Nash bargaining games. A unified cooperative Nash bargaining game-theoretic framework is proposed for the optimization problem of power control in DMRS, where IPCs are imposed to protect the communication user’s transmission and a minimum SINR requirement is employed to provide reliable target detection for each radar. Then, the existence and uniqueness of NBS are proven. In addition, an iterative Nash bargaining power control algorithm is presented, which converges quickly to a Pareto-optimal equilibrium for the cooperative game model.

### 3.2. Basics of Nash Bargaining Games

Let $M=\{1,2,\cdots ,M_{T}\}$ be a finite set of players, which denotes the radars in DMRS. Let $P_{i}$ be the power resource strategy of the player *i*, where $P_{i}=\left\{P_{i}|0\leq P_{i}\leq P_{i,\max},\sum_{i=1}^{M_{T}}P_{i}\leq P_{\mathrm{tot}}\right\}$. Let $U_{i}\left(P_{i},P_{-i}\right)$ be the utility function of player *i*, where $P_{-i}$ denotes the transmit power of all players apart from player *i*. The strategy space of the cooperative game model depends not only on the strategy of player *i*, but also on the strategies of all other players, i.e., $P=P_{1}\times P_{2}\times \cdots \times P_{M_{T}}$. Let $U_{i,\min}$ be the minimum utility requirement that player *i* expects. At this point, the cooperative game can be summarized as:

(10) $$\begin{array}{c} G=\langle M,{\left\{P_{i}\right\}}_{i\in M},{\left\{U_{i}\left(P_{i},P_{-i}\right)\right\}}_{i\in M}\rangle . \end{array}$$

In non-cooperative games, players do not cooperate with each other. The NE is the stable solution for a non-cooperative game, if the NE exists, and it is unique [36].

**Definition** **1.** *(NE): A pure-strategy NE in a non-cooperative game is defined as:*

(11) $$\begin{array}{c} U_{i}\left(P_{i}^{\mathrm{NE}},P_{-i}^{\mathrm{NE}}\right)\geq U_{i}\left(P_{i},P_{-i}^{\mathrm{NE}}\right),\;\;\forall P_{i}\in P_{i}, \end{array}$$

where $P_{i}^{\mathrm{NE}}$ denotes the transmit strategy of the *i*-th player in NE and $P_{-i}^{\mathrm{NE}}$ denotes the transmit strategy of the other $\left(M_{T}-1\right)$ players under NE except for player *i*. From (11), it is implied that a pure-strategy NE is the fixed point where no player can obtain a higher utility function value by changing its strategy unilaterally [1,36].

As previously mentioned, the NE in a cooperative game is not always efficient. For this reason, we resort to a cooperative Nash bargaining game [36]. Next, the definition of Pareto-optimal efficient point is given, where a player cannot find another point that improves the utility function values of all the players at the same time.

**Definition** **2.** (Pareto Optimality): A point is Pareto-optimal if and only if there is no other allocation that leads to superior performance for some players without causing inferior performance for some other players, that is, there exists no other allocation $U_{i}^{\prime }\left(P_{i},P_{-i}\right)$ such that $U_{i}^{\prime }\left(P_{i},P_{-i}\right)\geq U_{i}\left(P_{i},P_{-i}\right),\;\forall i\in M$, and $U_{i}^{\prime }\left(P_{i},P_{-i}\right)>U_{i}\left(P_{i},P_{-i}\right),\;\exists i\in M$ [36,37].

For a cooperative game model of multiple players, there may exist an infinite number of Pareto-optimal points [36]. Hence, we should investigate how to select a Pareto-optimal point for the cooperative game, in which a criterion is required to select the best Pareto-optimal point of the model. One of the possible criteria is the fairness of power resource allocation. To be specific, the fairness of the cooperative bargaining game model is NBS, which can provide a unique and fair Pareto-optimal point under Definition 3.

**Definition** **3.** $\bar{r}$ is an NBS in P for $U_{\min}=\left\{U_{1,\min},U_{2,\min},\cdots ,U_{M_{T},\min}\right\}$, i.e., $\bar{r}=H\left(P,U_{\min}\right)$, if the following axioms are satisfied [36,37]:

1) Individual rationality: $\bar{r_{i}}\geq U_{i,\min}$, where $\bar{r_{i}}\in \bar{r},\;\forall i\in M$.

2) Feasibility: $\bar{r}\in P$.

3) Pareto optimality: $\bar{r}$ is Pareto-optimal.

4) Independence of irrelevant alternatives: If $\bar{r}\in P^{\prime }\subset P$, $\bar{r}=H\left(P,U_{\min}\right)$, then $\bar{r}=H\left(P^{\prime },U_{\min}\right)$.

5) Independence of linear transformations: For any linear scale transformation ϱ, $\varrho \left(H\left(P,U_{\min}\right)\right)=H\left(\varrho \left(P\right),\varrho \left(U_{\min}\right)\right)$.

6) Symmetry: If P is invariant under all exchanges of players, that is $H_{i}\left(P,U_{\min}\right)=H_{j}\left(P,U_{\min}\right),\;\forall i,\;j$.

### 3.3. Utility Function Design and Power Control Game Formulation

**Theorem** **1.** A unique and fair NBS $P^{*}=\left\{P_{i}^{*},P_{-i}^{*}\right\}$ that satisfies all the axioms in Definition 3 can be obtained by maximizing a product term as follows:

(12) $$P^{*}=\arg\max_{P_{i}\in P_{i},\gamma_{i}\geq \gamma_{i,\min},\forall i\in M}\;\;\prod_{i=1}^{M_{T}}U_{i}\left(P_{i},P_{-i}\right)=\prod_{i=1}^{M_{T}}\left(\frac{\gamma_{i}-\gamma_{i,\min}}{\gamma_{i}}\right).$$

**Proof.**  The proof of Theorem 1 is omitted due to space limitations. A similar detailed proof can refer to [36,38]. ☐

Based on Theorem 1, the existence and uniqueness of NBS that satisfy all the axioms in Definition 3 can be proven.

The aim of our work is to maximize the radars’ utility functions while protecting communication user’s QoS. It is assumed that the interference power limit is sent by the communication system periodically. Therefore, the corresponding cooperative Nash bargaining game-theoretic power control problem for DMRS underlying the communication system can be formulated as:

(13a) $$\begin{array}{cc} P1:\;\;\; & \max_{{\left\{P_{i}\right\}}_{i\in M}}\;\;\prod_{i=1}^{M_{T}}U_{i}\left(P_{i},P_{-i}\right)=\prod_{i=1}^{M_{T}}{\left(\frac{\gamma_{i}-\gamma_{i,\min}}{\gamma_{i}}\right)}^{h_{i,i}^{t}}, \end{array}$$

(13b) $$\begin{array}{c} s.t.:\;\left\{\begin{array}{c} C1:\gamma_{i}\geq \gamma_{i,\min},\forall i\in M\\ C2:\sum_{i=1}^{M_{T}}g_{i}^{d}P_{i}\leq T_{\max}\\ C3:g_{i}^{d}P_{i}\leq T_{i,\max},\forall i\in M\\ C4:0\leq P_{i}\leq P_{i,\max},\forall i\in M\\ C5:\sum_{i=1}^{M_{T}}P_{i}\leq P_{\mathrm{tot}} \end{array}\right. \end{array}$$

where constraint C1 is imposed to guarantee that the target detection performance should be no smaller than a desired SINR threshold $\gamma_{i,\min}$; C2 and C3 set the global and individual tolerable interference levels, respectively; C4 limits the transmit power of each radar to be below $P_{i,\max}$; C5 stands for the total power constraint of the transmit power on DMRS. Note that one of the advantages of the designed utility function in P1 is that it results in player fairness [34]. It is indicated in [34,36] that a widely-utilized fairness metric is proportional fairness, which requires that $\prod_{i=1}^{M_{T}}U_{i}\left(P_{i},P_{-i}\right)=\prod_{i=1}^{M_{T}}\left(\frac{\gamma_{i}-\gamma_{i,\min}}{\gamma_{i}}\right)\geq 0$ for the interested utility $\gamma_{i}\;\forall i\in M$. Moreover, introducing the balancing factor $h_{i,i}^{t}$ can further guarantee the fairness among different players located at different sites [32,34].

**Lemma** **1.** Define $V_{i}\left(P_{i},P_{-i}\right)≜\ln\left(U_{i}\left(P_{i},P_{-i}\right)\right)=h_{i,i}^{t}\ln\left(\frac{\gamma_{i}-\gamma_{i,\min}}{\gamma_{i}}\right),\;i\in M$. These objective functions are concave and injective, which satisfy all the Nash axioms in Definition 3.

**Proof.**  As we can observed, the utility functions $U_{i}\left(P_{i},P_{-i}\right)$ are continuous with respect to $P_{i}$. Then, taking the second derivative of $U_{i}\left(P_{i},P_{-i}\right)$ with respect to $P_{i}$, we get:

(14) $$\begin{array}{c} \frac{\partial U_{i}\left(P_{i},P_{-i}\right)}{\partial P_{i}}=-\frac{2h_{i,i}^{t}\gamma_{i,\min}}{P_{i}^{2}{\left(\gamma_{i}-\gamma_{i,\min}\right)}^{2}}\left(\gamma_{i}-\frac{1}{2}\gamma_{i,\min}\right)>0. \end{array}$$

Thus, $U_{i}\left(P_{i},P_{-i}\right)$ is concave in $P_{i}$. Subsequently, $V_{i}\left(P_{i},P_{-i}\right)=\ln\left(U_{i}\left(P_{i},P_{-i}\right)\right)$ is also concave in $P_{i}$. If $U_{i}\left(P_{i},P_{-i}\right)$ is injective, then $V_{i}\left(P_{i},P_{-i}\right)$ is concave. Therefore, $V_{i}\left(P_{i},P_{-i}\right)$ defined above satisfies all the axioms required by Definition 3 and Theorem 1. ☐

According to Theorem 1 and Lemma 1, the unique Nash bargaining equilibrium with fairness can be found over the strategy space. Then, taking advantage of the increasing property of the logarithmic function, the optimization problem P1 can be rewritten as:

(15a) $$\begin{array}{cc} P2:\;\;\; & \max_{{\left\{P_{i}\right\}}_{i\in M}}\;\;\sum_{i=1}^{M_{T}}V_{i}\left(P_{i},P_{-i}\right)=\sum_{i=1}^{M_{T}}h_{i,i}^{t}\ln\left(\frac{\gamma_{i}-\gamma_{i,\min}}{\gamma_{i}}\right), \end{array}$$

(15b) $$\begin{array}{c} s.t.:\;\left\{\begin{array}{c} C1:\gamma_{i}\geq \gamma_{i,\min},\forall i\in M\\ C2:\sum_{i=1}^{M_{T}}g_{i}^{d}P_{i}\leq T_{\max}\\ C3:g_{i}^{d}P_{i}\leq T_{i,\max},\forall i\in M\\ C4:0\leq P_{i}\leq P_{i,\max},\forall i\in M\\ C5:\sum_{i=1}^{M_{T}}P_{i}\leq P_{\mathrm{tot}} \end{array}\right. \end{array}$$

### 3.4. Potential Extension

For brevity, we concentrate on a single communication system case in this study. However, the calculations and results can be extended to the multiple communication systems case, in which the IPCs are imposed to protect each communication user’s QoS. For the *Q* communication users scenario, the resulting problem is reformulated as:

(16a) $$\begin{array}{cc} P3:\;\;\; & \max_{{\left\{P_{i}\right\}}_{i\in M}}\;\;\sum_{i=1}^{M_{T}}V_{i}\left(P_{i},P_{-i}\right)=\sum_{i=1}^{M_{T}}h_{i,i}^{t}\ln\left(\frac{\gamma_{i}-\gamma_{i,\min}}{\gamma_{i}}\right), \end{array}$$

(16b) $$\begin{array}{c} s.t.:\;\left\{\begin{array}{c} C1:\gamma_{i}\geq \gamma_{i,\min},\forall i\in M\\ C2:\sum_{i=1}^{M_{T}}g_{i,q}^{d}P_{i}\leq T_{q,\max},\forall q\in Q\\ C3:g_{i,q}^{d}P_{i}\leq T_{i,q,\max},\forall i\in M,\forall q\in Q\\ C4:0\leq P_{i}\leq P_{i,\max},\forall i\in M\\ C5:\sum_{i=1}^{M_{T}}P_{i}\leq P_{\mathrm{tot}} \end{array}\right. \end{array}$$

where all the parameters with subscript *q* denote the corresponding ones of communication user q(q∈Q={1,2,⋯,Q}). Then, we can also employ the following iterative procedure to search for the optimal power control results for P3. In this scenario, the proposed power control scheme can be extended to the multiple communication users case by adding the IPCs for each user.

## 4. Nash Bargaining Power Control Solutions for DMRS

### 4.1. Solution of the Cooperative Game

Herein, we derive the unique equilibrium by solving the constrained optimization problem in (15) utilizing the method of Lagrange multipliers [32]. Introducing Lagrange multipliers ${\left\{\eta_{i}\right\}}_{i=1}^{M_{T}}$, ϕ, ${\left\{\xi_{i}\right\}}_{i=1}^{M_{T}}$, ${\left\{\mu_{i}\right\}}_{i=1}^{M_{T}}$, ${\left\{\psi_{i}\right\}}_{i=1}^{M_{T}}$ and κ for the multiple constraints, the Lagrangian of problem (15) can equivalently be solved by maximizing the following expression:

(17) $$\begin{array}{c} J\left({\left\{P_{i}\right\}}_{i=1}^{M_{T}},{\left\{\eta_{i}\right\}}_{i=1}^{M_{T}},\phi ,{\left\{\xi_{i}\right\}}_{i=1}^{M_{T}},{\left\{\mu_{i}\right\}}_{i=1}^{M_{T}},{\left\{\psi_{i}\right\}}_{i=1}^{M_{T}},\kappa \right)\\ =\sum_{i=1}^{M_{T}}h_{i,i}^{t}\ln\left(\frac{\gamma_{i}-\gamma_{i,\min}}{\gamma_{i}}\right)-\eta_{i}\left(\gamma_{i}-\gamma_{i,\min}\right)+\phi \left(\sum_{i=1}^{M_{T}}g_{i}^{d}P_{i}-T_{\max}\right)+\xi_{i}\left(g_{i}^{d}P_{i}-T_{i,\max}\right)\\ -\psi_{i}P_{i}+\kappa \left(\sum_{i=1}^{M_{T}}P_{i}-P_{\mathrm{tot}}\right). \end{array}$$

Based on the standard optimization methods and the Karush–Kuhn–Tucker (KKT) conditions, the transmit power allocation for radar *i* can be obtained by taking the first derivative of (17) with respect to $P_{i}$, which is expressed as follows:

(18) $$\begin{array}{cc} \frac{\partial J}{\partial P_{i}} & =h_{i,i}^{t}\frac{\gamma_{i}}{\gamma_{i}-\gamma_{i,\min}}\frac{\frac{\partial \gamma_{i}}{\partial P_{i}}\gamma_{i,\min}}{\gamma_{i}^{2}}-\eta_{i}\frac{\partial \gamma_{i}}{\partial P_{i}}+\phi g_{i}^{d}+\xi_{i}g_{i}^{d}+\mu_{i}-\psi_{i}+\kappa . \end{array}$$

Substituting $\frac{\partial \gamma_{i}}{\partial P_{i}}=\frac{\gamma_{i}}{P_{i}}$ into (18), we get:

(19) $$\begin{array}{cc} \frac{\partial J}{\partial P_{i}} & =h_{i,i}^{t}\frac{\gamma_{i}}{\gamma_{i}-\gamma_{i,\min}}\frac{\frac{\gamma_{i}}{P_{i}}\gamma_{i,\min}}{\gamma_{i}^{2}}-\eta_{i}\frac{\gamma_{i}}{P_{i}}+\left(\phi +\xi_{i}\right)g_{i}^{d}+\mu_{i}-\psi_{i}+\kappa \\ & =h_{i,i}^{t}\frac{\gamma_{i,\min}}{P_{i}\left(\gamma_{i}-\gamma_{i,\min}\right)}-\eta_{i}\frac{\gamma_{i}}{P_{i}}+\left(\phi +\xi_{i}\right)g_{i}^{d}+\mu_{i}-\psi_{i}+\kappa . \end{array}$$

Letting $\frac{\partial J}{\partial P_{i}}=0$ and substituting $\gamma_{i}=\frac{h_{i,i}^{t}P_{i}}{I_{-i}}$ into (19), we can get:

(20) $$\begin{array}{cc} & P_{i}\left(\frac{h_{i,i}^{t}P_{i}}{I_{-i}}-\gamma_{i,\min}\right)=\frac{h_{i,i}^{t}\gamma_{i,\min}}{\eta_{i}\frac{\gamma_{i}}{P_{i}}-\left(\phi +\xi_{i}\right)g_{i}^{d}-\mu_{i}+\psi_{i}-\kappa }\\ ⟹ & \frac{h_{i,i}^{t}}{I_{-i}}P_{i}^{2}-\gamma_{i,\min}P_{i}-\frac{h_{i,i}^{t}\gamma_{i,\min}}{\eta_{i}\frac{\gamma_{i}}{P_{i}}-\left(\phi +\xi_{i}\right)g_{i}^{d}-\mu_{i}+\psi_{i}-\kappa }=0. \end{array}$$

After basic algebraic manipulations, we get the following optimal solution:

(21) $$\begin{array}{c} P_{i}^{*}=\frac{1}{2}\left(\frac{I_{-i}}{h_{i,i}^{t}}\gamma_{i,\min}+\sqrt{A^{*}}\right), \end{array}$$

where:

(22) $$\begin{array}{c} A^{*}=\frac{I_{-i}^{2}}{{\left(h_{i,i}^{t}\right)}^{2}}\gamma_{i,\min}^{2}+\frac{4\gamma_{i,\min}I_{-i}^{2}}{h_{i,i}^{t}\eta_{i}^{*}+\left[\psi_{i}^{*}-\left(\phi^{*}+\xi_{i}^{*}\right)g_{i}^{d}-\mu_{i}^{*}-\kappa^{*}\right]I_{-i}}, \end{array}$$

and the subscript ${\left(\cdot \right)}^{*}$ represents optimality.

In this work, we employ the fixed-point technique to derive an iterative procedure that updates the transmit power control decisions, which can be given as:

(23) $$\begin{array}{c} P_{i}^{\left(ite+1\right)}={\left[\frac{1}{2}\left(\frac{P_{i}^{\left(ite\right)}}{\gamma_{i}^{\left(ite\right)}}\gamma_{i,\min}+\sqrt{B^{\left(ite\right)}}\right)\right]}_{0}^{P_{i,\max}}, \end{array}$$

where:

(24) $$\begin{array}{c} B^{\left(ite\right)}={\left(\frac{P_{i}^{\left(ite\right)}}{\gamma_{i}^{\left(ite\right)}}\right)}^{2}\gamma_{i,\min}^{2}+\frac{4\gamma_{i,\min}h_{i,i}^{t}{\left(\frac{P_{i}^{\left(ite\right)}}{\gamma_{i}^{\left(ite\right)}}\right)}^{2}}{\eta_{i}^{\left(ite\right)}+\left[\psi_{i}^{\left(ite\right)}-\left(\phi^{\left(ite\right)}+\xi_{i}^{\left(ite\right)}\right)g_{i}^{d}-\mu_{i}^{\left(ite\right)}-\kappa^{\left(ite\right)}\right]\left(\frac{P_{i}^{\left(ite\right)}}{\gamma_{i}^{\left(ite\right)}}\right)}, \end{array}$$

${\left[x\right]}_{a}^{b}=\max\left\{\min\left(x,b\right),a\right\}$, and ite denotes the iteration index.

### 4.2. Update of the Lagrange Multipliers

The Lagrange multipliers ${\left\{\eta_{i}^{\left(ite\right)}\right\}}_{i=1}^{M_{T}}$, $\phi^{\left(ite\right)}$, ${\left\{\xi_{i}^{\left(ite\right)}\right\}}_{i=1}^{M_{T}}$, ${\left\{\mu_{i}^{\left(ite\right)}\right\}}_{i=1}^{M_{T}}$, ${\left\{\psi_{i}^{\left(ite\right)}\right\}}_{i=1}^{M_{T}}$ and $\kappa^{\left(ite\right)}$ need to be updated to guarantee the fast convergence property. Several effective approaches can be employed in the update of Lagrange multipliers. In this paper, the sub-gradient technique is utilized to update the multipliers, as formulated as follows:

(25) $$\left\{\begin{array}{cc} \eta_{i}^{\left(ite+1\right)} & ={\left[\eta_{i}^{\left(ite\right)}-\beta^{\left(ite\right)}\left(\gamma_{i}^{\left(ite+1\right)}-\gamma_{i,\min}\right)\right]}^{+},\\ \phi^{\left(ite+1\right)} & ={\left[\phi^{\left(ite\right)}-\beta^{\left(ite\right)}\left(T_{\max}-\sum_{i=1}^{M_{T}}g_{i}^{d}P_{i}^{\left(ite+1\right)}\right)\right]}^{+},\\ \xi_{i}^{\left(ite+1\right)} & ={\left[\xi_{i}^{\left(ite\right)}-\beta^{\left(ite\right)}\left(T_{i,\max}-g_{i}^{d}P_{i}^{\left(ite+1\right)}\right)\right]}^{+},\\ \mu_{i}^{\left(ite+1\right)} & ={\left[\mu_{i}^{\left(ite\right)}-\beta^{\left(ite\right)}\left(P_{i,\max}-P_{i}^{\left(ite+1\right)}\right)\right]}^{+},\\ \psi_{i}^{\left(ite+1\right)} & ={\left[\psi_{i}^{\left(ite\right)}-\beta^{\left(ite\right)}P_{i}^{\left(ite+1\right)}\right]}^{+},\\ \kappa^{\left(ite+1\right)} & ={\left[\kappa^{\left(ite\right)}-\beta^{\left(ite\right)}\left(P_{\mathrm{tot}}-\sum_{i=1}^{M_{T}}P_{i}^{\left(ite+1\right)}\right)\right]}^{+}, \end{array}\right.$$

where ${\left(x\right)}^{+}=\max\left(0,x\right)$, β denotes the step size of iteration $ite\;(ite\in \left\{1,2,\cdots ,L_{\max}\right\})$ and $L_{\max}$ denotes the maximum number of iterations. It should be mentioned that the Lagrange multipliers ${\left\{\eta_{i}^{\left(ite\right)}\right\}}_{i=1}^{M_{T}}$, ${\left\{\xi_{i}^{\left(ite\right)}\right\}}_{i=1}^{M_{T}}$, ${\left\{\mu_{i}^{\left(ite\right)}\right\}}_{i=1}^{M_{T}}$, and ${\left\{\psi_{i}^{\left(ite\right)}\right\}}_{i=1}^{M_{T}}$ are locally updated, while $\phi^{\left(ite\right)}$ and $\kappa^{\left(ite\right)}$ are updated through the cooperation of different players.

### 4.3. Iterative Nash Bargaining Power Control Algorithm

Based on the derivations and analyses in Section 3.1 and Section 3.2, a distributed algorithm is presented as an implementation of the cooperative power control solution. The convergence of Algorithm 1 is ensured by utilizing the sub-gradient approach.

**Complexity analysis.** The computational complexity of Algorithm 1 is dominated by the procedure of sub-gradient iteration steps and the size of DMRS. In Algorithm 1, the calculation of (23) for each radar in DMRS entails $M_{T}$ operations in each iteration. Suppose a sub-gradient method employed in Algorithm 1 needs Δ iterations to converge; updating ${\left\{\eta_{i}^{\left(ite\right)}\right\}}_{i=1}^{M_{T}}$, ${\left\{\xi_{i}^{\left(ite\right)}\right\}}_{i=1}^{M_{T}}$, ${\left\{\mu_{i}^{\left(ite\right)}\right\}}_{i=1}^{M_{T}}$ and ${\left\{\psi_{i}^{\left(ite\right)}\right\}}_{i=1}^{M_{T}}$ needs $O(M_{T})$ operations each, and the computation of $\phi^{\left(ite\right)}$ and $\kappa^{\left(ite\right)}$ calls O(1) operation each; thus, Δ is a polynomial function of $M_{T}^{4}$. The total computational complexity of Algorithm 1 is $O(M_{T}\Delta )$. Moreover, as demonstrated by the simulations in Section 4, the convergence can be obtained after 10 iterations.

**Implementation overhead.** In the iterative procedure, Step 3(c) requires radar cooperation to update the Lagrange multipliers $\phi^{\left(ite\right)}$ and $\kappa^{\left(ite\right)}$ for the total transmit power constraint and IPC, respectively, whereas the power control decisions are made locally. Therefore, Algorithm 1 is distributed, and the practicality is ensured.

**Algorithm 1** Cooperative bargaining power control algorithm.
1:Initialize $\gamma_{i,\min}$, $T_{\max}$, $T_{i,\max}$ and Lagrange multipliers ${\left\{\eta_{i}^{\left(ite\right)}\right\}}_{i=1}^{M_{T}}$, $\phi^{\left(ite\right)}$, ${\left\{\xi_{i}^{\left(ite\right)}\right\}}_{i=1}^{M_{T}}$, ${\left\{\mu_{i}^{\left(ite\right)}\right\}}_{i=1}^{M_{T}}$, ${\left\{\psi_{i}^{\left(ite\right)}\right\}}_{i=1}^{M_{T}}$ and $\kappa^{\left(ite\right)}$; set ite=1;   2:Initialize ${\left\{P_{i}^{\left(ite\right)}\right\}}_{i=1}^{M_{T}}$ with a uniform transmit power allocation among all radars;3:**repeat**  **for** i=1 to $M_{T}$, **do**   (a) Radars update ${\left\{P_{i}^{\left(ite\right)}\right\}}_{i=1}^{M_{T}}$ according to (23);   (b) Radars update ${\left\{\eta_{i}^{\left(ite\right)}\right\}}_{i=1}^{M_{T}}$, ${\left\{\xi_{i}^{\left(ite\right)}\right\}}_{i=1}^{M_{T}}$, ${\left\{\mu_{i}^{\left(ite\right)}\right\}}_{i=1}^{M_{T}}$, and ${\left\{\psi_{i}^{\left(ite\right)}\right\}}_{i=1}^{M_{T}}$ according to (25);  **end for**  (c) The fusion centre updates $\phi^{\left(ite\right)}$ and $\kappa^{\left(ite\right)}$ according to (25) and broadcasts those values to all radars via the backhaul link; set ite←ite+1;4:**until** Convergence or $ite=L_{\max}$5:**return** ${\left\{P_{i}^{\left(ite\right)}\right\}}_{i=1}^{M_{T}}$


## 5. Simulation Results and Discussion

### 5.1. Simulation Settings

**Figure 2:**
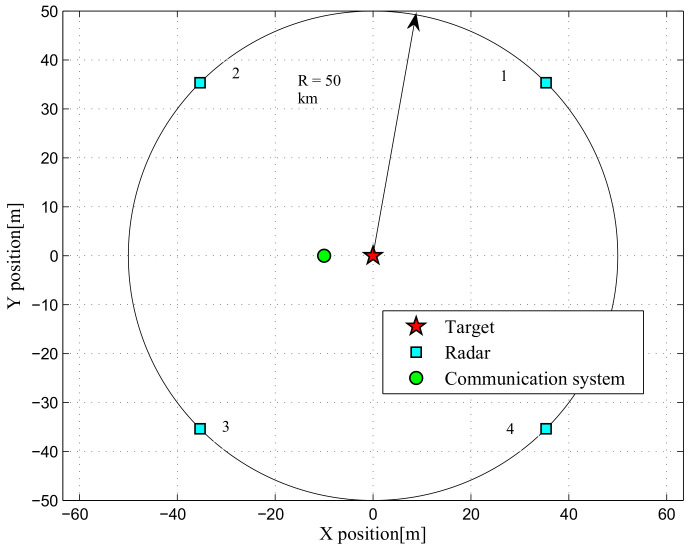
Simulated 2D scenario with locations of multiple radars, communication user and target.

In this section, the simulation results are provided to verify the performance of our presented scheme. The simulations regard a DMRS of $P_{\mathrm{tot}}=4000\;W$ consisting of $M_{T}=4$ radars. The locations of multiple radars, the communication user and target are depicted in Figure 2. To evaluate the influence of the geometry between target and DMRS on the power control results, we consider two different target locations. In the first case, we assume that the target is located at [0,0]km, while the target is located at $[-25/\sqrt{2},25/\sqrt{2}]\;\mathrm{km}$ in the second case. Moreover, in order to better shed light on the effects of several factors on the power control results, we consider two target RCS models $\sigma_{\mathrm{RCS},1}=\left[1,1,1,1\right]m^{2}$ and $\sigma_{\mathrm{RCS},2}=\left[4,2,1,30\right]m^{2}$.

In our simulation, the number of received pulses in every time slot is N=512. The maximum number of iterations is set to be $L_{\max}=30$. Other system parameters for our scenario are summarized in Table 1. The desired probabilities of false alarm and target detection are $P_{i,\mathrm{FA}}=10^{-6}$ and $P_{i,D}=\;0.99\left(\forall i\right)$, respectively. Then, the detection threshold can be obtained as $\lambda_{i}=0.027$ for each radar, and the corresponding SINR threshold is $\gamma_{i,\min}=10\;\mathrm{dB}\left(\forall i\right)$. In addition, it is worth mentioning that all the parameters, such as radar transmit powers, target detection requirement and the locations of multiple radars, communication user and target, can be set as other values, which only affect the Nash equilibrium results.

### 5.2. Simulation Results

#### 5.2.1. Convergence Performance

**Figure 3:**
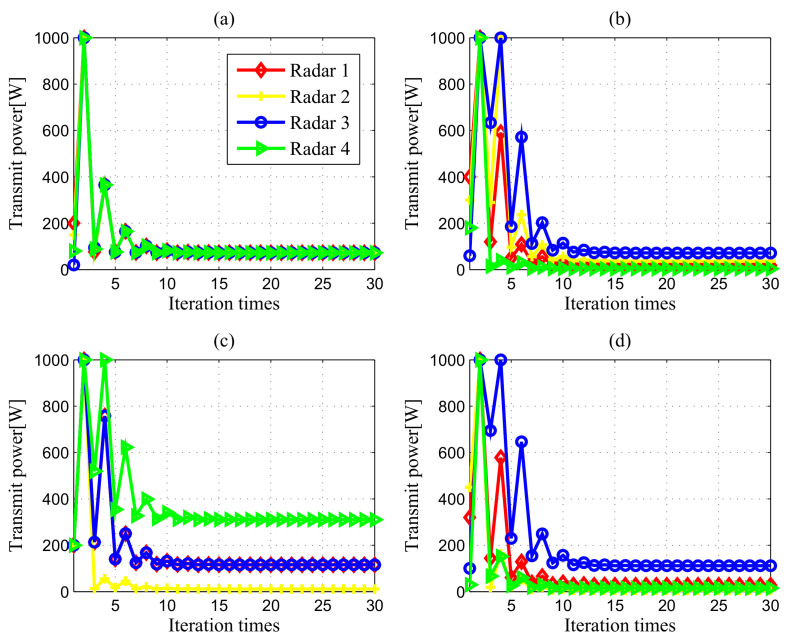
Convergence of power control results in different cases: (**a**) Case 1 with $\sigma_{\mathrm{RCS},1}$; (**b**) Case 1 with $\sigma_{\mathrm{RCS},2}$; (**c**) Case 2 with $\sigma_{\mathrm{RCS},1}$; (**d**) Case 2 with $\sigma_{\mathrm{RCS},2}$.

Figure 3 portrays the transmit power iterations of all the radars in DMRS for different initial powers, where the Nash bargaining game is initialized with P=[200,150,20,80]W, P=[400,300,60,180]W, P=[200,200,200,200]W and P=[350,280,100,10]W, respectively. As can be observed from Figure 3, the transmit power of each radar converges after 10 iterations. Certainly, the choice of the Lagrange multipliers is crucial to the convergence behaviour. It should be noted that the convergence values of the transmit powers are not dependent on the choice of the Lagrangian multipliers. The different Lagrangian multipliers only affect the convergence speed of the proposed algorithm. Here, since the feasible power requirements are quite harsh, we initialize with $\eta_{i}^{\left(0\right)}=10$, $\phi_{i}^{\left(0\right)}=10$, $\xi_{i}^{\left(0\right)}=10$, $\mu_{i}^{\left(0\right)}=10$, $\psi_{i}^{\left(0\right)}=10$, $\kappa_{i}^{\left(0\right)}=10\left(\forall i\right)$. These results, together with previous analysis, indicate that our proposed NBS-based power control algorithm converges to the Pareto-optimal equilibrium, implying that it is robust to feasible initial transmit power allocation.

**Figure 4:**
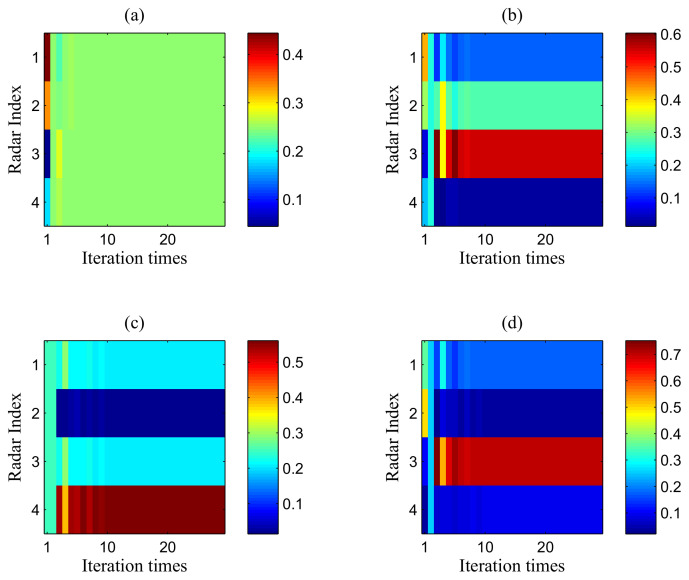
The power ratio results in different cases: (**a**) Case 1 with $\sigma_{\mathrm{RCS},1}$; (**b**) Case 1 with $\sigma_{\mathrm{RCS},2}$; (**c**) Case 2 with $\sigma_{\mathrm{RCS},1}$; (**d**) Case 2 with $\sigma_{\mathrm{RCS},2}$.

Furthermore, the transmit power ratio results in different cases employing the proposed algorithm are highlighted in Figure 4, where the transmit power ratio is defined as:

(26) $$\tau_{i}=\frac{P_{i}}{\sum_{i=1}^{M_{T}}P_{i}}.$$

In Figure 4, the coloured areas actually represent the ratio of the transmit power of each radar. To be specific, in Figure 4a, the transmit power is distributed uniformly to each radar, which is because the target’s RCS with respect to each radar and the range between target and radar node are the same. In Figure 4b,d, more power is allocated to Radar 3 to maintain the predefined target detection threshold, which is due to the fact that the target RCS with respect to Radar 3 is much smaller than the other radars. On the other hand, it can be seen from Figure 4c that, since Radar 2 is the closest to the target, the least transmit power is allocated to Radar 2. In other words, the radars farther from the target tend to be allocated more transmit power. Therefore, it can be concluded that power resources are distributed to the radars that have weaker propagation channel gains in the iterative process. Among these radars, a larger amount of transmit power is allocated to the radars that have a relatively larger distance and/or smaller target RCS.

**Figure 5:**
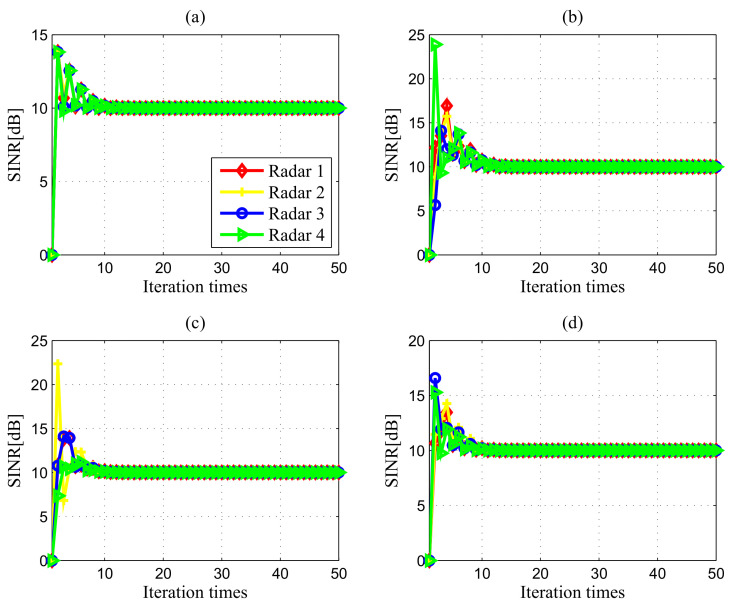
Convergence of SINR in different cases: (**a**) Case 1 with $\sigma_{\mathrm{RCS},1}$; (**b**) Case 1 with $\sigma_{\mathrm{RCS},2}$; (**c**) Case 2 with $\sigma_{\mathrm{RCS},1}$; (**d**) Case 2 with $\sigma_{\mathrm{RCS},2}$.

Results in Figure 5 illustrate the SINR convergence curves of the proposed algorithm. Obviously, the SINR converges fast with 10–15 iterations required to reach a steady state. As is expected, each radar only seeks to reach the specified SINR requirement via iterative power control without necessarily reaching the maximum SINR values of the cooperative NBS-based power control framework [1].

#### 5.2.2. Superiority of Our Proposed Algorithm

**Figure 6:**
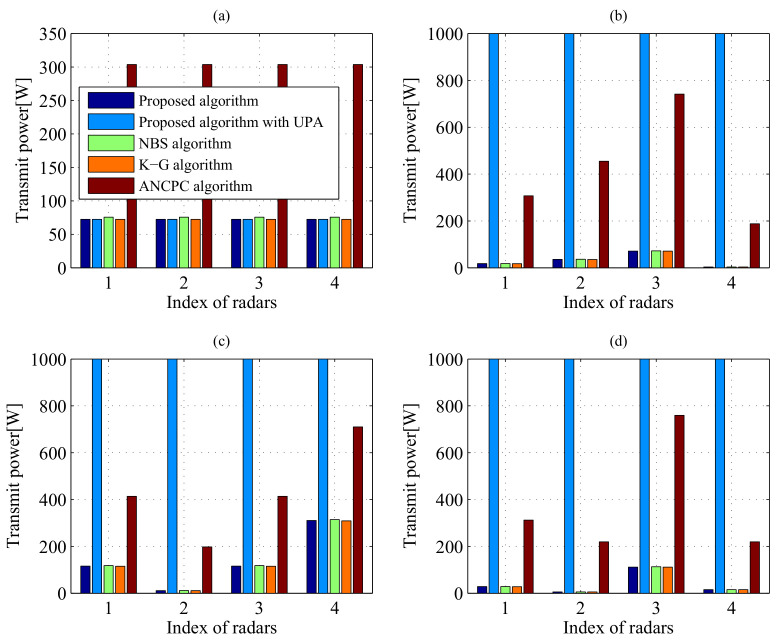
Comparisons of equilibrium transmit power in different cases employing various methods: (**a**) Case 1 with $\sigma_{\mathrm{RCS},1}$; (**b**) Case 1 with $\sigma_{\mathrm{RCS},2}$; (**c**) Case 2 with $\sigma_{\mathrm{RCS},1}$; (**d**) Case 2 with $\sigma_{\mathrm{RCS},2}$.

**Figure 7:**
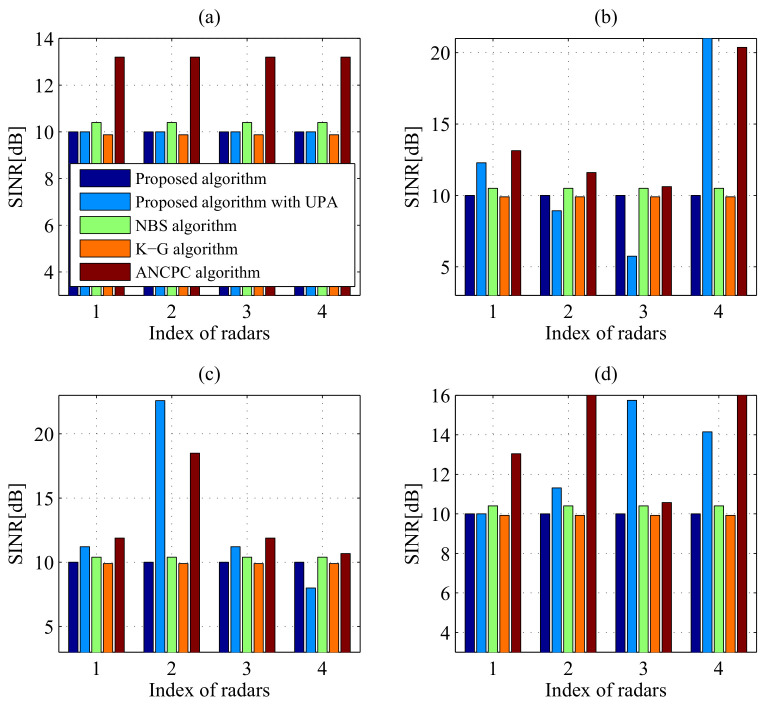
Comparisons of equilibrium SINR in different cases employing various methods: (**a**) Case 1 with $\sigma_{\mathrm{RCS},1}$; (**b**) Case 1 with $\sigma_{\mathrm{RCS},2}$; (**c**) Case 2 with $\sigma_{\mathrm{RCS},1}$; (**d**) Case 2 with $\sigma_{\mathrm{RCS},2}$.

Figure 6 and Figure 7 compare the transmit power levels and SINR performance of five power control algorithms: (1) the proposed algorithm; (2) the proposed algorithm with uniform power allocation (UPA); (3) the traditional NBS-based power control algorithm; (4) the Koskie and Gajic (K-G) algorithm [39]; (5) the adaptive non-cooperative power control (ANCPC) algorithm [40]. It should be noted that the traditional NBS-based method, K-G algorithm and ANCPC algorithm ignore the IPCs (8) and (9), which optimize transmit power allocation in DMRS without consideration of harmful interference to the communication system. From Figure 6 and Figure 7, the proposed algorithm outperforms the traditional NBS-based power control algorithm, which transmits more power than our presented approach. In Figure 6, it should be pointed out that the K-G algorithm transmits the least power, while from Figure 7, it can be seen that not all the SINR values can reach the desired SINR threshold. On the other hand, the ANCPC approach consumes the most power and achieves the highest SINR level, which is due to the fact that each player behaves in a selfish and rational manner to maximize its own utility function. In Figure 6b–d, for the proposed algorithm with UPA, the DMRS cannot perceive the interference environment well and accordingly makes the most appropriate transmit power control decision. Thus, although the specified SINR threshold is satisfied, it leads to larger transmit power consumption than the proposed power control algorithm. To conclude, the proposed algorithm not only minimizes the total transmit power consumption of DMRS, but also ensures the desired target detection thresholds of all radars in a spectrum sharing environment.

#### 5.2.3. Spectrum Sharing Performance

**Figure 8:**
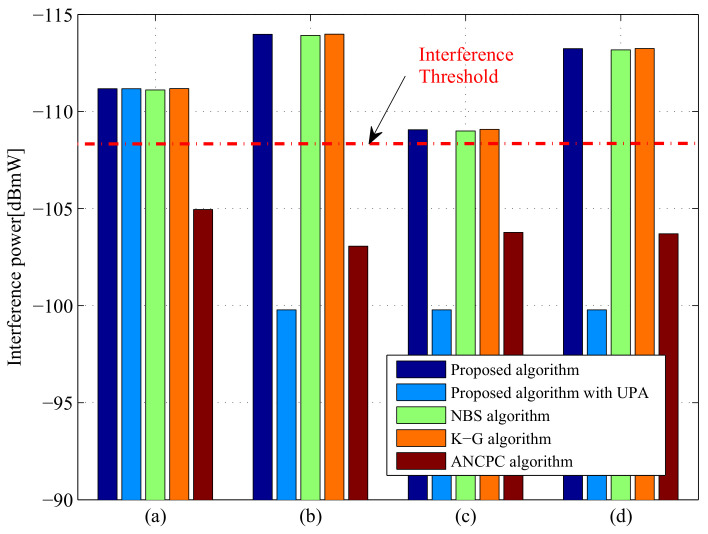
Comparisons of interference power levels in different cases employing various methods: (**a**) Case 1 with $\sigma_{\mathrm{RCS},1}$; (**b**) Case 1 with $\sigma_{\mathrm{RCS},2}$; (**c**) Case 2 with $\sigma_{\mathrm{RCS},1}$; (**d**) Case 2 with $\sigma_{\mathrm{RCS},2}$.

The comparison of our proposed algorithm and the other four state-of-the-art power control approaches in terms of received interference power at the communication user for different scenarios is given in Figure 8. From Figure 8, one interesting observation is that the received interference power levels at the communication user for our proposed algorithm, NBS algorithm and K-G algorithm are below the predetermined maximum acceptable interference power threshold $T_{\max}$ for the communication system, which are considerably lower than those of the proposed algorithm with UPA and the ANCPC algorithm. Consequently, the QoS can be satisfied by ensuring that the DMRS does not generate high harmful interference for the communication system. However, as indicated in [32], the traditional NBS method and K-G method are not ideal, which is due to the fact that the traditional NBS method consumes more power than the proposed algorithm, while the SINR requirement of each radar cannot be maintained when utilizing the K-G method. In conclusion, the DMRS can share the spectrum with wireless communication systems only when the interference power generated by DMRS is strictly controlled, which should be below the maximum acceptable interference power level of the communication user.

## 6. Conclusion Remarks

In this paper, we have studied the problem of power control for DMRS underlying a wireless communication system. The resulting power control problem was developed as a cooperative Nash bargaining game model, in which an interference power limit is imposed to protect the communication user and a minimum SINR requirement is employed to provide reliable target detection for each radar. Then, the existence, uniqueness and fairness of the NBS to the cooperative game model were proven. Furthermore, a cooperative Nash bargaining power control method with low computational complexity and fast convergence was developed, and it was shown to converge to a Pareto-optimal equilibrium for the cooperative game model. Finally, numerical results revealed the superiority of the proposed algorithm over other existing approaches in terms of power consumption, target detection and spectrum coexistence performance between DMRS and the communication system in the same frequency band. It was also demonstrated that the proposed algorithm not only converges within a few iterations, but also achieves good performance with significant reduction on the implementation overhead, illustrating its potential for a practical design.

Our future work is to investigate the problem of cooperative game-theoretic power control for the coexisting DMRS and multiple wireless communication systems. Different game-theoretic model-based power control for DMRS in a spectrum sharing environment will be part of our future work, and novel utility functions may be designed for DMRS and communication users.

## Figures and Tables

**Table 1:** Coexisting DMRS and wireless communication system parameters.

Parameter	Value	Parameter	Value
$G_{t}$	27dB	$G_{r}$	27dB
$G_{t}^{{}^{\prime }}$	−30dB	$G_{r}^{\prime }$	−30dB
$G_{c}$	0dB	$\sigma_{n}^{2}$	$10^{-18}\;W$
$T_{\max}$	−108dBmW	$c_{i,j}$	0.01
λ	0.10m	$P_{i,\max}\left(\forall i\right)$	1000W
